# Supplementary material for: Allosteric interactions prime androgen receptor dimerization and activation
Source: Mol Cell. Author manuscript; Available in PMC 2023 Jun 2. (PMC9177810; doi:10.1016/j.molcel.2022.03.035)
Supplement: 1 — Figures S1-13, Table S1 (Separate PDF) [file NIHMS1795365-supplement-1.pdf]

Supplementary Data

Wasmuth, *et al.*

Allosteric interactions prime androgen receptor dimerization and activation

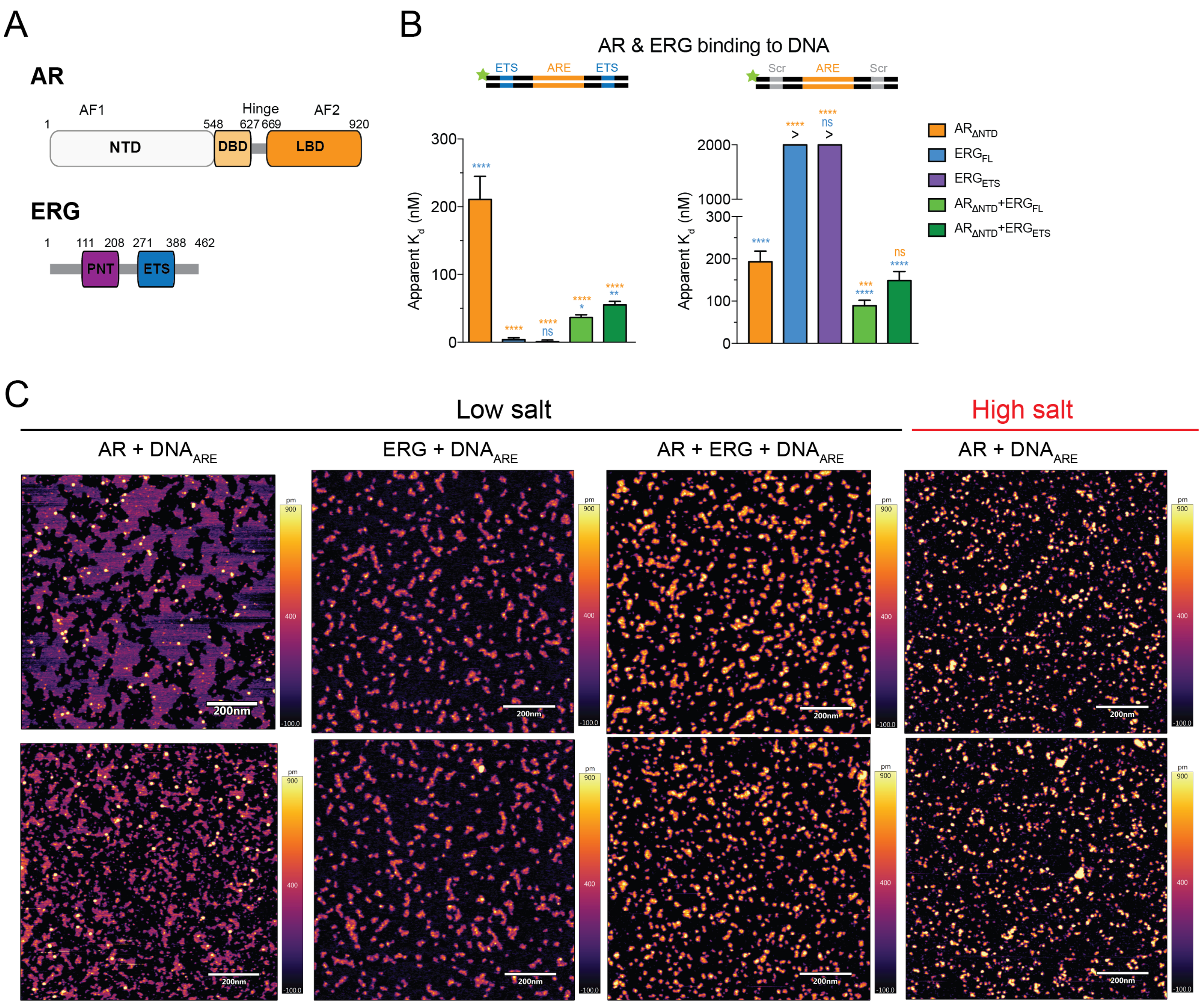

Figure S1. ERG chaperones AR to facilitate DNA binding, Related to Figure 1. (A) Domain structures of AR and ERG. (B) Fluorescence polarization of recombinant AR lacking its N-terminus (ΔNTD AR) and ERG variants on consensus ARE DNA with (left) or without (right) ETS motifs. (C) Additional atomic force microscopy (AFM) images from Main Figure 1A showing solubilizing and stabilizing effects of ERG on ΔNTD AR bound to DNA in low salt.

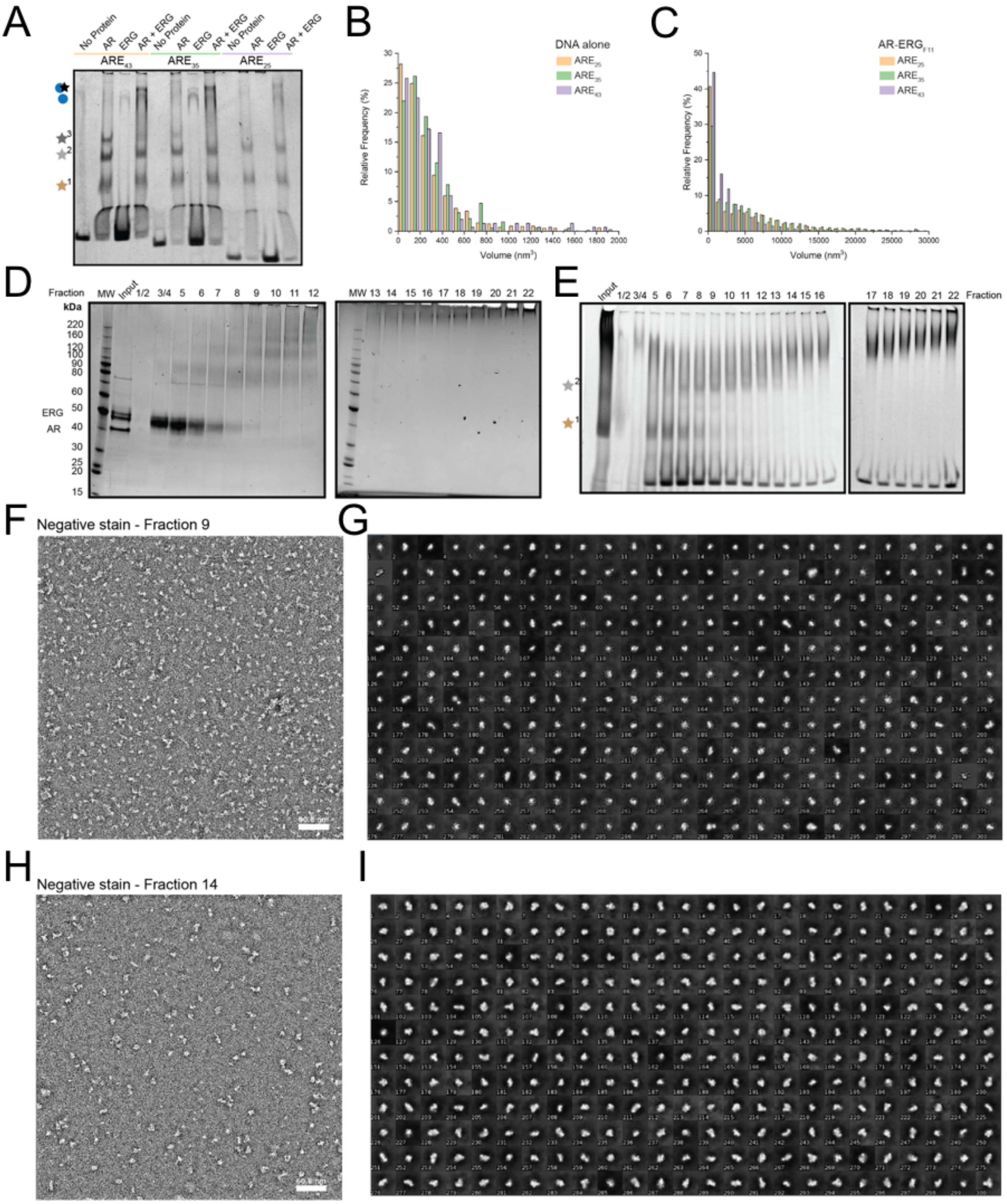
A
No Protein
AR
ERG
AR + ERG
ARE43
ARE35
ARE25
B
DNA alone
Relative Frequency (%)
Volume (nm3)
C
AR-ERGF11
D
Fraction
kDa
MW
Input
ERG
AR
E
Fraction
F
Negative stain - Fraction 9
G
H
Negative stain - Fraction 14
I

Figure S2

Figure S2. Isolation and trapping of a DNA-bound AR-ERG ternary complex, Related to Figure 1. (A) DNA gel shift of 50 nM unlabeled palindromic ARE duplex DNA lacking an ETS consensus sequence with 250 nM indicated recombinant protein. DNA substrates were 43 (ARE_{43}), 35 (ARE_{35}), or 25 (ARE_{25}) bp in length. 4-20% TBE PAGE stained with Sybr Gold. Stars and circles represent AR- and ERG-shifted species, respectively. (B, C) Histogram summarizing volume distribution obtained by AFM of DNA alone (B) or the indicated DNA-bound AR-ERG complex corresponding to Grafix fraction 11 (C, see below). (D, E) Grafix fractions of the ARE_{35}-bound AR-ERG complex migrating through a 5-20% sucrose gradient and used for subsequent cryo-EM studies. ARE_{35} was chosen for further structural analyses as the results in (A-C) suggest that this length is the shortest to promote complex formation while preventing higher order multimerization, and exhibits the least heterogeneity by AFM. (D) Total protein gels. 4-12% Bis-Tris PAGE stained with Sypro Ruby. (E) DNA gel. 4-20% TBE PAGE stained with Sybr Gold. F-I) Screening of Grafix fractions by negative stain EM show variable size distribution and sample heterogeneity. (F, G) Representative negative stain micrograph and 2D class averages of smaller MW Grafix fraction 9. A mixture of free subunits and cross-linked species can be observed. (H, I) Representative negative stain micrograph and 2D class averages of larger MW Grafix fraction 14. A large proportion of classes represent higher order oligomers due to over cross-linking.

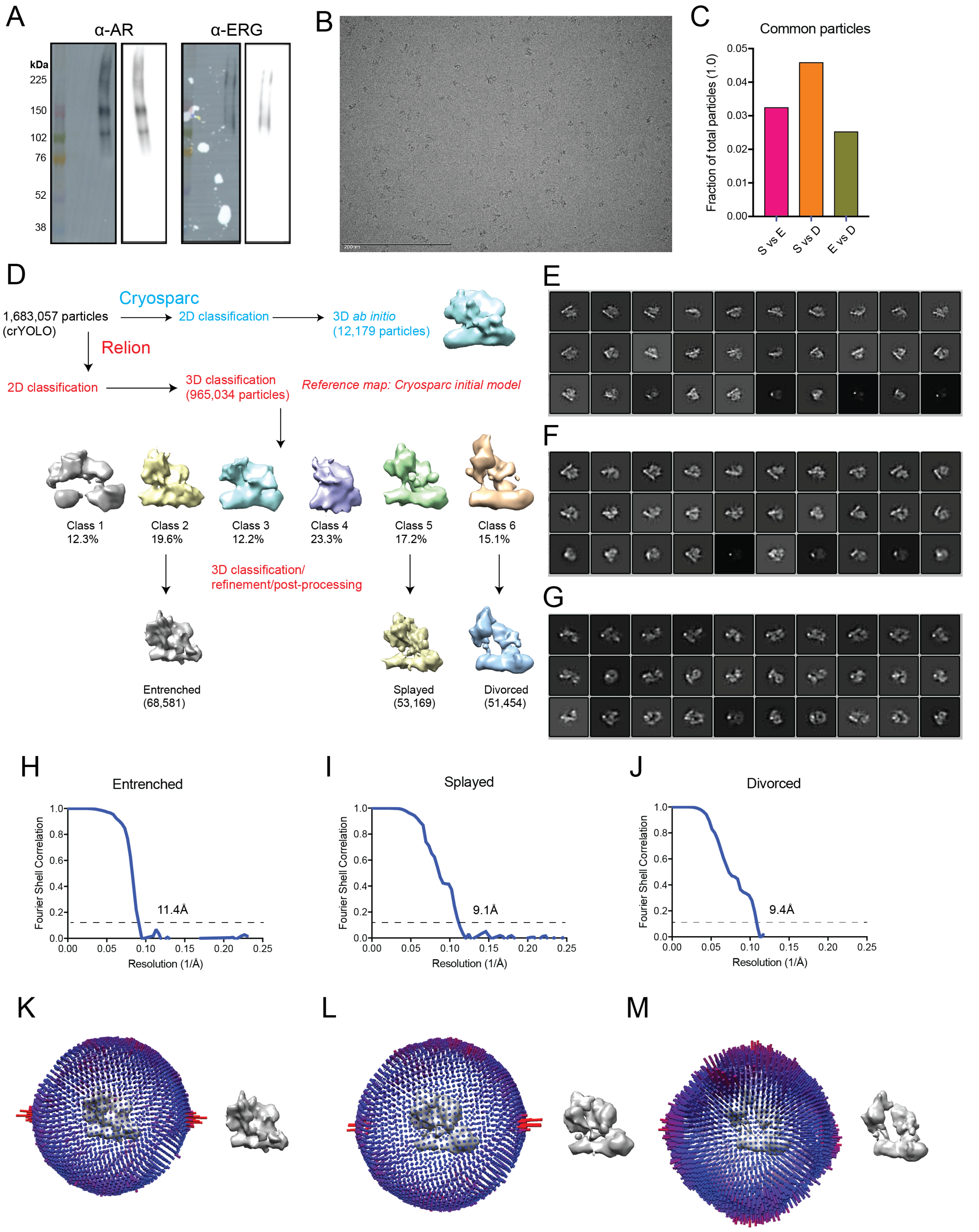
A
α-AR
α-ERG
kDa
225
150
102
76
52
38
B
C
Common particles
Fraction of total particles (1.0)
0.05
0.04
0.03
0.02
0.01
0.00
S vs E
S vs D
E vs D
D
Cryosparc
1,683,057 particles
(crYOLO)
2D classification
3D ab initio
(12,179 particles)
Relion
2D classification
3D classification
(965,034 particles)
Reference map: Cryosparc initial model
Class 1
12.3%
Class 2
19.6%
Class 3
12.2%
Class 4
23.3%
Class 5
17.2%
Class 6
15.1%
3D classification/
refinement/post-processing
Entrenched
(68,581)
Splayed
(53,169)
Divorced
(51,454)
E
F
G
H
Entrenched
Fourier Shell Correlation
11.4Å
Resolution (1/Å)
I
Splayed
9.1Å
J
Divorced
9.4Å
K
L
M

Figure S3. Single particle cryo-EM analysis of the DNA-bound AR-ERG ternary complex, Related to Figure 1. (A) AR and ERG immunoblots of AR-ERG complex bound to ARE_{35} used for cryo-EM studies (Grafix fraction 11 from Figure S2D, E) showing distribution of AR- and ERG-crosslinked species in the final preparation. Both AR and ERG are present in the mono cross-linked species (~100 kDa). (B) Representative cryo-EM micrograph. (C) Number of particles shared between the Splayed, Entrenched, and Divorced states. Shared particles represented as fraction of total particle count in each 3D model. (D) Single particle cryo-EM data processing workflow. (E-G) 2D classification of particle stacks for the (E) Entrenched, (F) Splayed, and (G) Divorced models. H-J) FSC curves and reported resolutions for the (H) Entrenched, (I) Splayed, and (J) Divorced states. K-M) Euler angle and distribution plots and corresponding 3D model for (K) Entrenched, (I) Splayed, and (M) Divorced models. Side, top, back, and bottom views are shown.

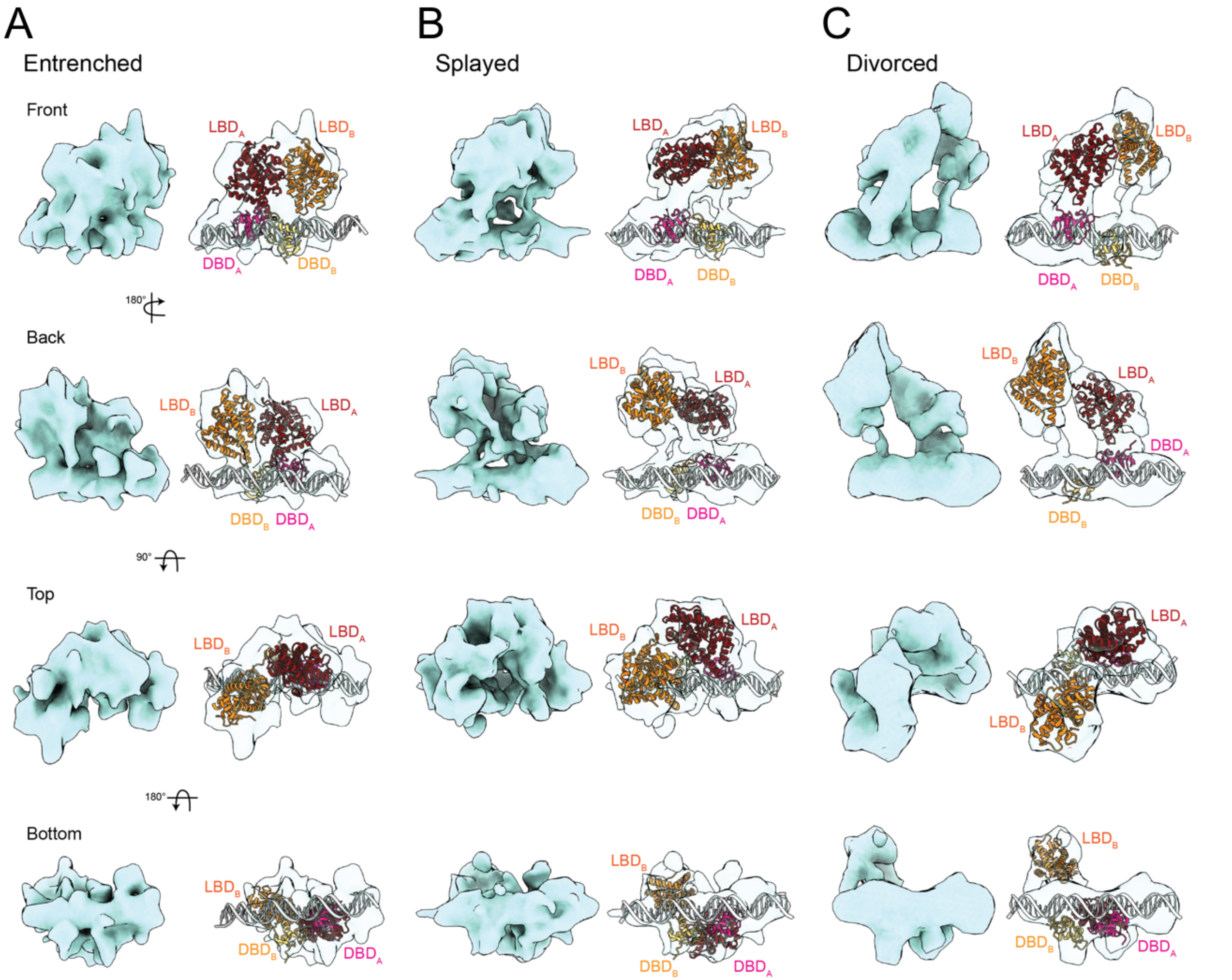

Figure S4. Docking of X-ray coordinates into cryo-EM maps of AR bound to DNA, Related to Figure 1. Cryo-EM electron density of the (A) Entrenched, (B) Splayed, and (C) Divorced conformations of AR bound to DNA are shown as blue surfaces in opaque (left) or transparent (right) representations. Right: individually docked domains of X-ray coordinates using AR LBD monomers (He *et al.*, 2004) (PBD: 1XOW, red/orange) and the DBD dimer bound to ARE repeat DNA (Shaffer *et al.*, 2004) (PDB: 1R4I, yellow/pink).

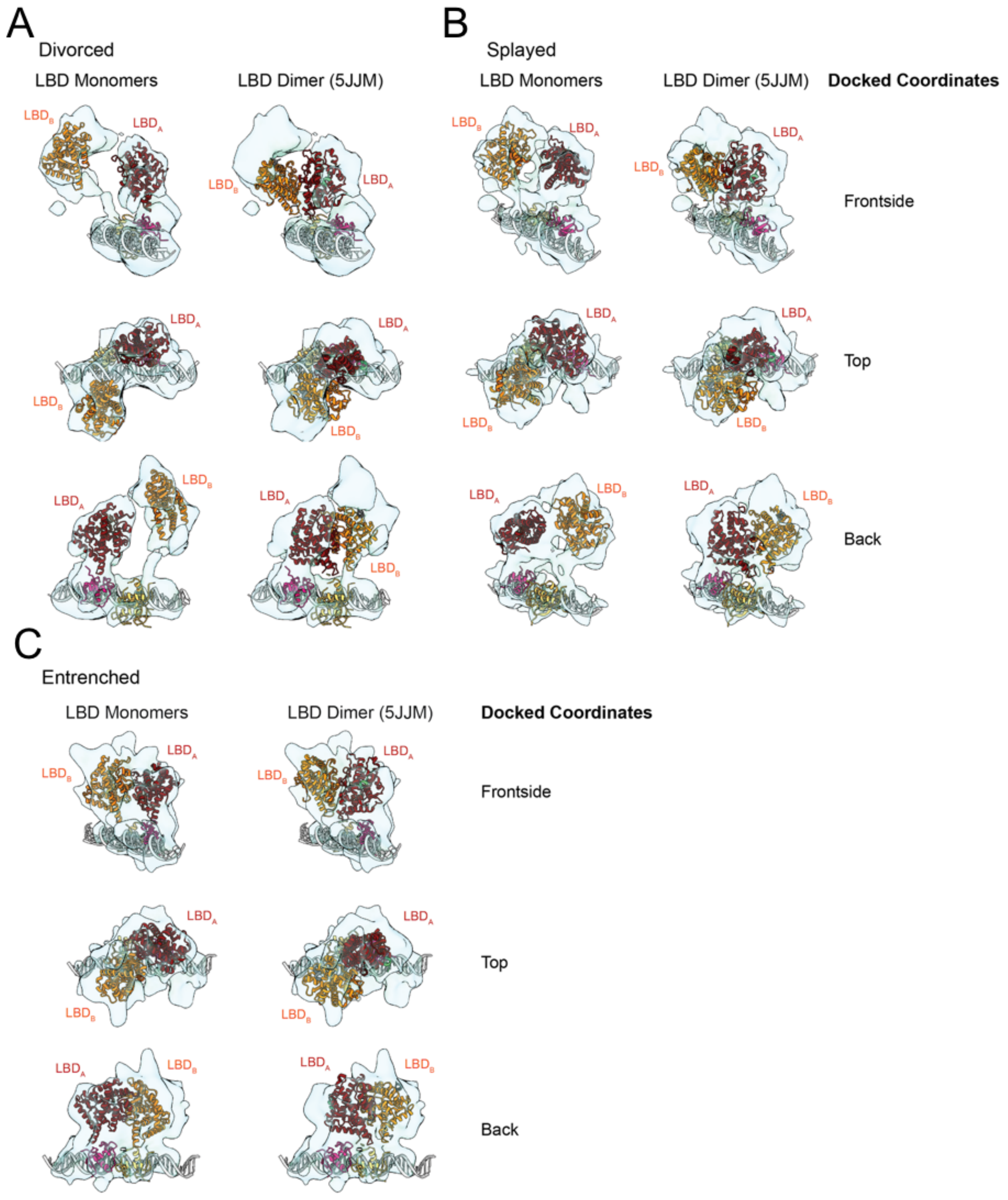

Figure S5. Plasticity among the LBD-LBD dimer interface, Related to Figure 1. Cryo-EM models with indicated X-ray coordinates docked show similarity and divergence from the crystal structure of the AR LBD dimer crosslinked proximal to the dimer interface at residue C686 (PDB: 5JJM) (Nadal *et al.*, 2017), with the Divorced and Splayed models showing the most deviation. Left: X-ray coordinates of LBD monomer (PBD: 1XOW) (He *et al.*, 2004) individually docked into the density of the (A) Divorced, (B) Splayed, and (C) Entrenched states. Right: Similar docking strategy for the X-ray coordinates of 1 unit of the LBD dimer (PDB: 5JJM).

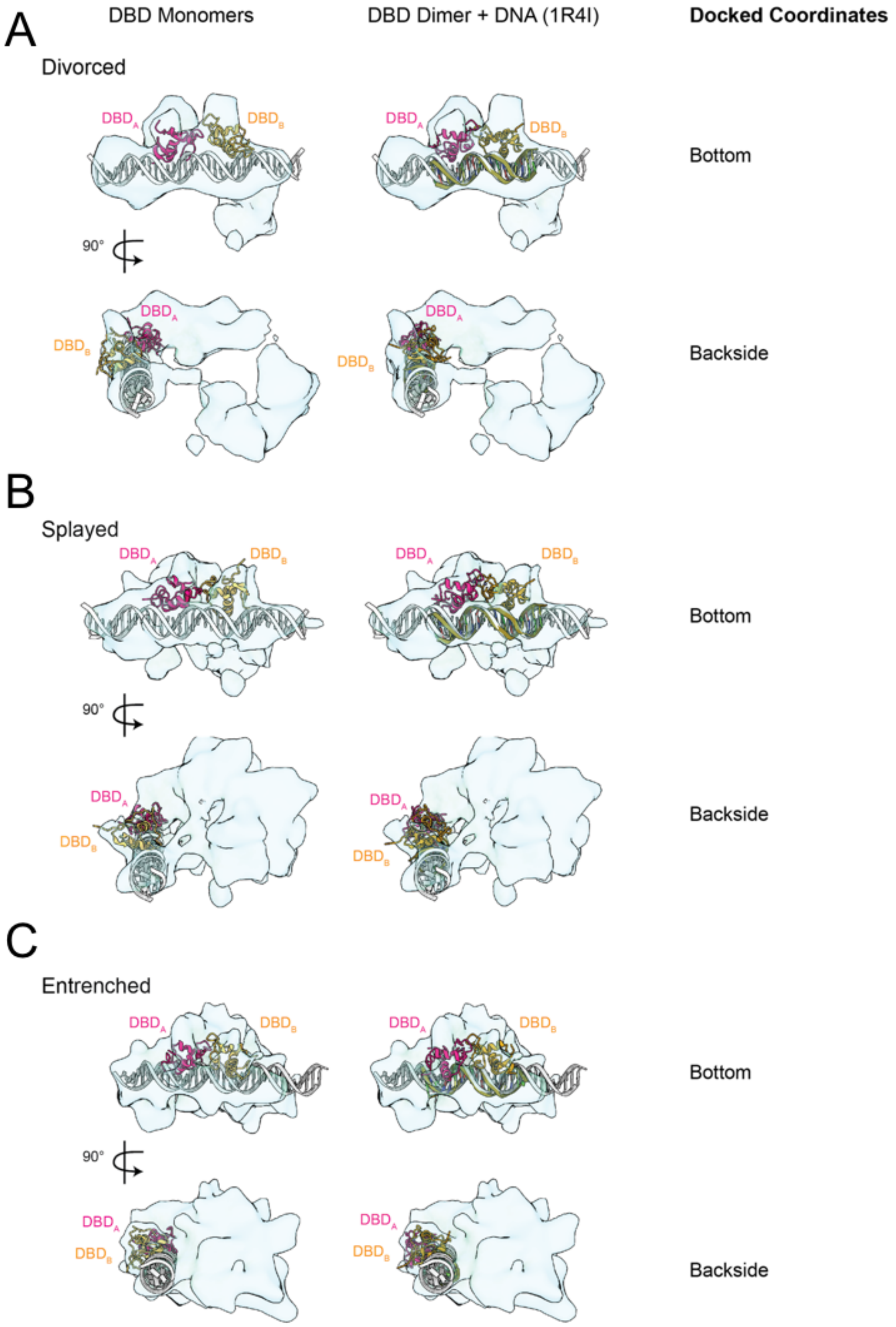

Figure S6. Plasticity among the DBD-DBD dimer interface, Related to Figure 1. Cryo-EM models with indicated X-ray coordinates docked show similarity and divergence from the crystal structure of the AR DBD dimer bound to two direct repeats of the AR hexameric half site (Shaffer *et al.*, 2004), with the Splayed and Divorced models showing increasing deviation, respectively, while DBD dimer aligns well with the density from the Entrenched model. Left: X-ray coordinates of DBD monomers (PBD: 1R4I) individually docked into the density of the (A) Divorced, (B) Splayed, and (C) Entrenched states. Right: Similar docking strategy using the DBD dimer from the X-ray structure (PDB: 1R4I).

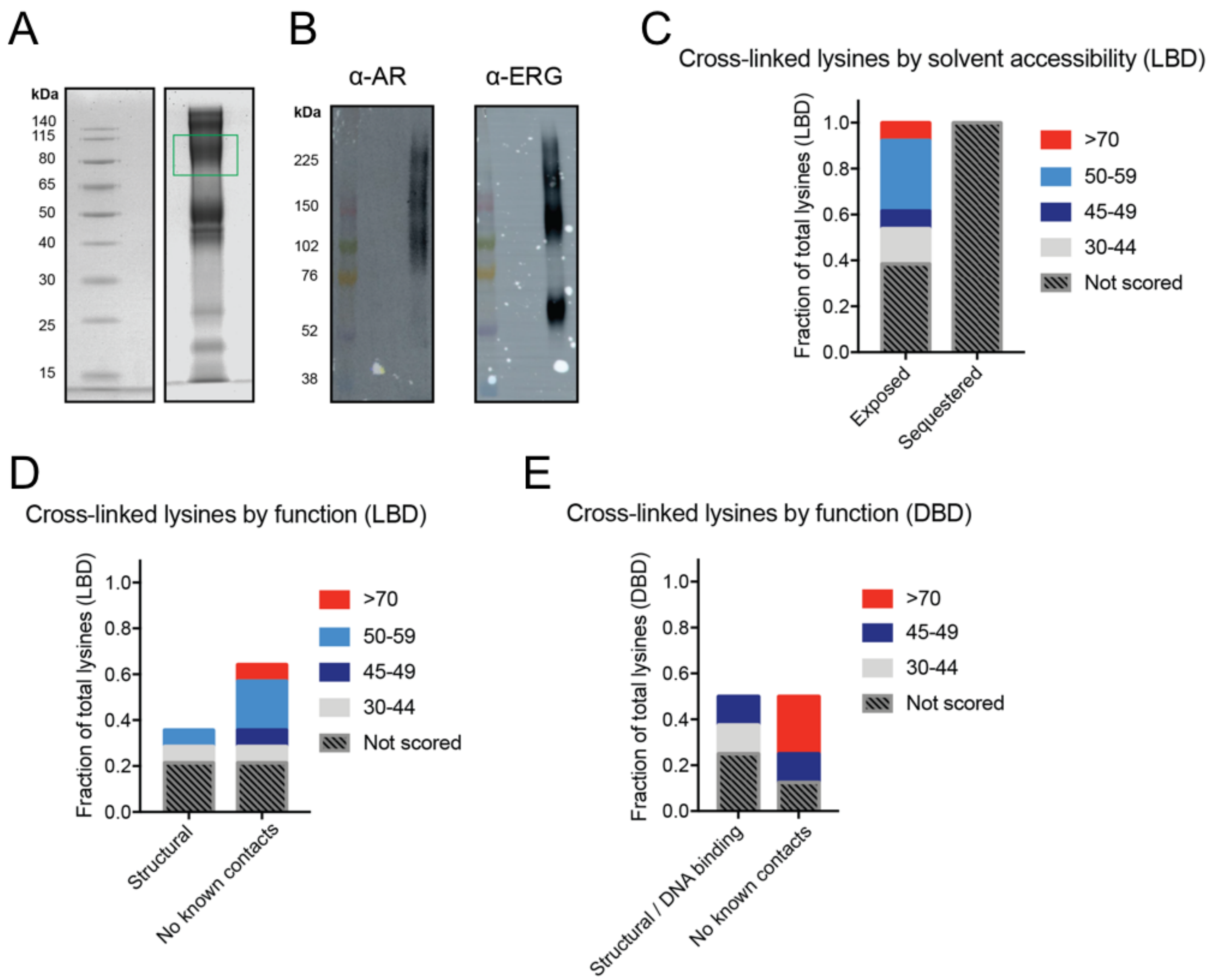

Figure S7. Characterization of DSSO cross-linked species, Related to Figure 2. (A) Coomassie-stained total protein gel of DSSO cross-linked AR-ERG complex bound to ARE_{35} DNA and separated by ultracentrifugation (Fraction 11). Boxed fragments were excised and analyzed by XL-MS. (B) AR and ERG immunoblots of sample in (A) shows the distribution of free and cross-linked AR and ERG. A cutoff score of 45 was applied to define rational cross-links, with 87% of scored total lysines free of any known structural or DNA binding contacts. Breakdowns visualized in panels C-E. (C) Solvent exposed or sequestered lysines within the AR LBD that underwent DSSO cross-linking and their corresponding scores. (D) Stratification of lysines within the AR LBD based on their participation in structural contacts, and their corresponding cross-linking scores. (E) Similar analysis as in (D) for the AR DBD. Lysines were deemed functional if not involved in either structural or DNA binding contacts. No cross-links were detected to lysines of the DNA Recognition helix (Shaffer *et al.*, 2004), and no

cross-links scored 45 or above were to lysines involved in any known DNA binding contacts, suggesting this scoring threshold represents productive cross-links.

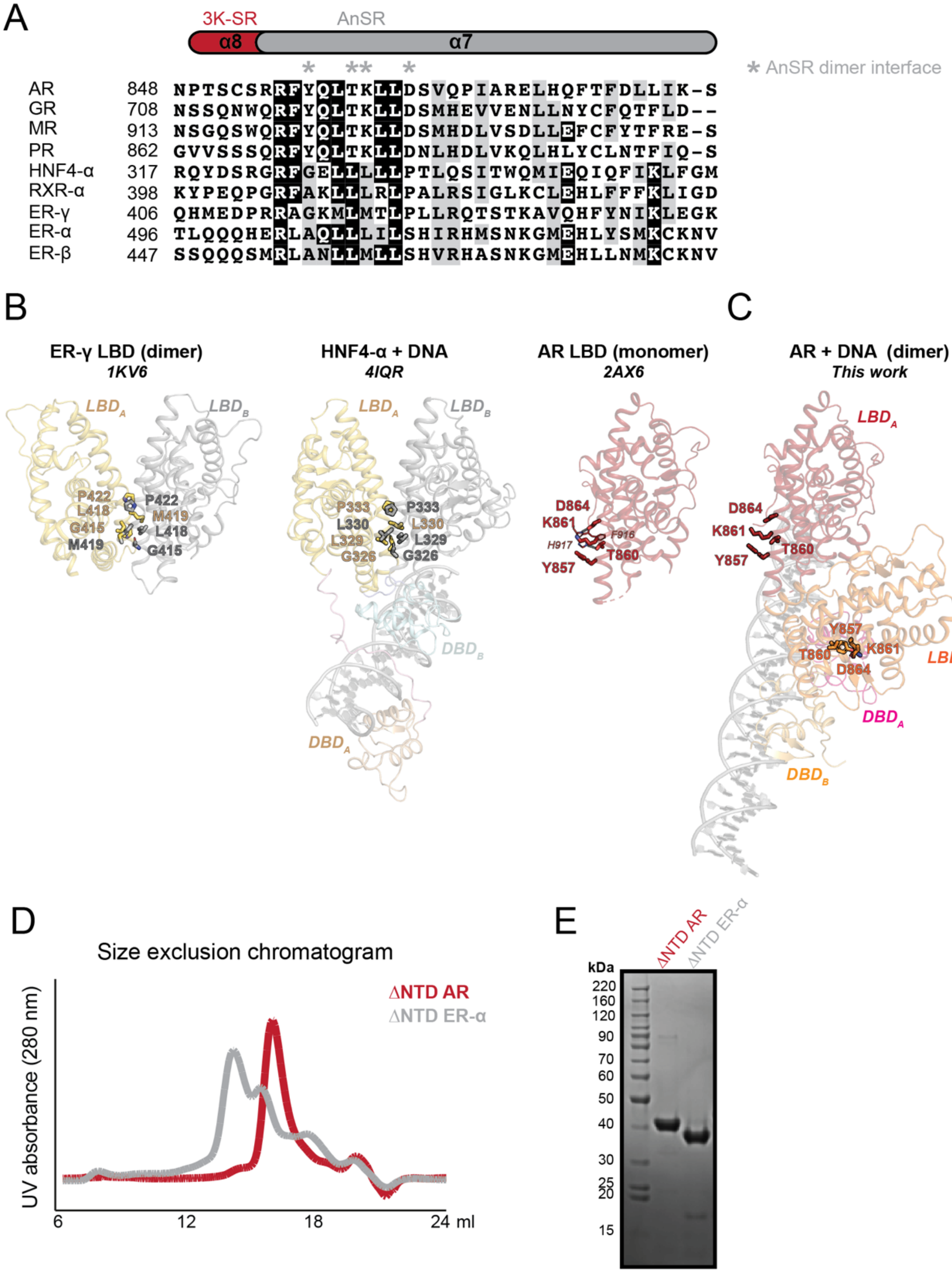
A
3K-SR
AnSR
α8
α7
* AnSR dimer interface
AR 848 NPTSCSRRFYQLTKLLDSVQPIARELHQFTFDLLIK-S
GR 708 NSSQNWQRFYQLTKLLDSMHEVVENLLNYCFQTFLD--
MR 913 NSGQSWQRFYQLTKLLDSMHDLVSDLLEFCFYTFRE-S
PR 862 GVVSSSQRFYQLTKLLDNLHDLVKQLHLYCLNTFIQ-S
HNF4-α 317 RQYDSRGRFGELLLLLPTLQSITWQMIEQIQFIKLFGM
RXR-α 398 KYPEQPGRFAKLLLRLPALRSIGLKCLEHLFFFKLIGD
ER-γ 406 QHMEDPRRAGKMLMTLPLLRQTSTKAVQHFYNIKLEGK
ER-α 496 TLQQQHERLAQLLLILSHIRHMSNKGMEHLYSMKCKNV
ER-β 447 SSQQQSMRLANLLMLLSHVRHASNKGMEHLLNMKCKNV
B
ER-γ LBD (dimer)
1KV6
HNF4-α + DNA
4IQR
AR LBD (monomer)
2AX6
C
AR + DNA (dimer)
This work
GR LBD (dimer)
1M2Z
D
Size exclusion chromatogram
ΔNTD AR
ΔNTD ER-α
UV absorbance (280 nm)
6
12
18
24 ml
E
ΔNTD AR
ΔNTD ER-α
kDa
220
160
120
90
70
60
50
40
30
25
20
15

Figure S8

Figure S8. The AR dimer interface is distinct from that of the ancestral and type II nuclear receptors, Related to Figure 2. (A) Sequence alignment of the dimerization helix of the ancestral steroid receptors (AnSR) and type II NR family compared to the 3-ketosteroid receptors (3K-SR). Residues implicated in the AnSR and type II NR dimer interfaces are hydrophobic or nonpolar, and indicated with asterisks; the corresponding residues in 3K-SR family members are polar or charged. Alignment generated by Clustal Omega and BoxShade (Edgar, 2004). (B) AnSR and type II NR dimer interface residues mapped onto the crystal structures of the dimeric form of the ER-γ LBD (PDB: 1KV6, left) (Greschik *et al.*, 2002), and HNF4-α bound to DNA (PDB: 4IQR, middle) (Chandra *et al.*, 2013), and the AR LBD monomer (PDB: 1XOW) (He *et al.*, 2004). (C) AnSR / type II NR dimer interface residues are solvent-exposed and distal from the 3K-SR dimer interface in the cryo-EM structures of AR bound to DNA (this work, Entrenched model shown) and the dimeric form of the GR LBD (PDB: 1M2Z) (Bledsoe *et al.*, 2002). These residues either make structural contacts with the 3K-SR-specific C-terminal extension (italics) or are implicated in cooperative interactions between the LBD and DBD, i.e. K861 (Main Figure 2B, C). (D, E) ∆NTD AR does not constitutively dimerize. (D) Comparison of size exclusion chromatograms (Superdex 200) between AR and the corresponding N-terminal truncation of the AnSR ER-α. The LBD of ER-α has been previously shown to dimerize (Hochberg *et al.*, 2020), and forms higher molecular weight (MW) oligomers by size exclusion chromatography, despite having a similar MW as AR by SDS-PAGE (E). In contrast, AR elutes at a volume consistent with its monomeric form.

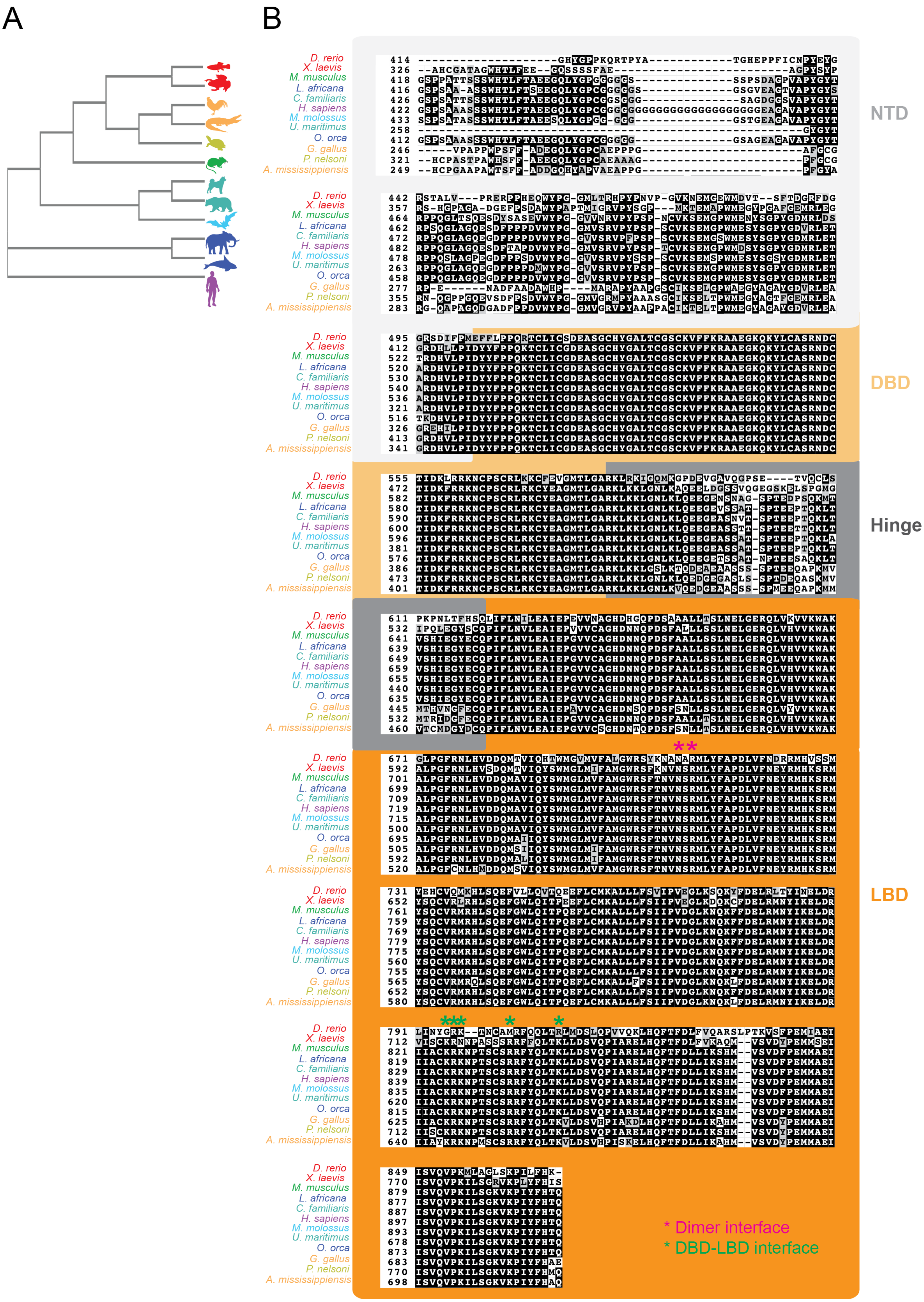
A
B
D. rerio
X. laevis
M. musculus
L. africana
C. familiaris
H. sapiens
M. molossus
U. maritimus
O. orca
G. gallus
P. nelsoni
A. mississippiensis
NTD
DBD
Hinge
LBD
* Dimer interface
* DBD-LBD interface

Figure S9

Figure S9. Conservation of surfaces implicated in AR interdomain cooperativity, Related to Figure 2. (A) AR phylogenetic tree, with *H. sapiens* in purple, and most divergent species in red (*D. rerio* and *X. laevis*). Orange: *G. gallus* and *A. mississippiensis*. Yellow-green: *P. nelsoni*. Green: *M. musculus*. Teal: *C. familiaris* and *U. maritimus*. Light blue: *M. molossus*. Indigo: *L. africana* and *O. orca*. (B) Sequence alignment, with NTD shaded in light grey, DBD in yellow-orange, Hinge in dark grey, and LBD in orange. LBD residues bridging Dimer and DBD interfaces are indicated with asterisks. Analyses performed with Clustal Omega and BoxShade (Edgar, 2004).

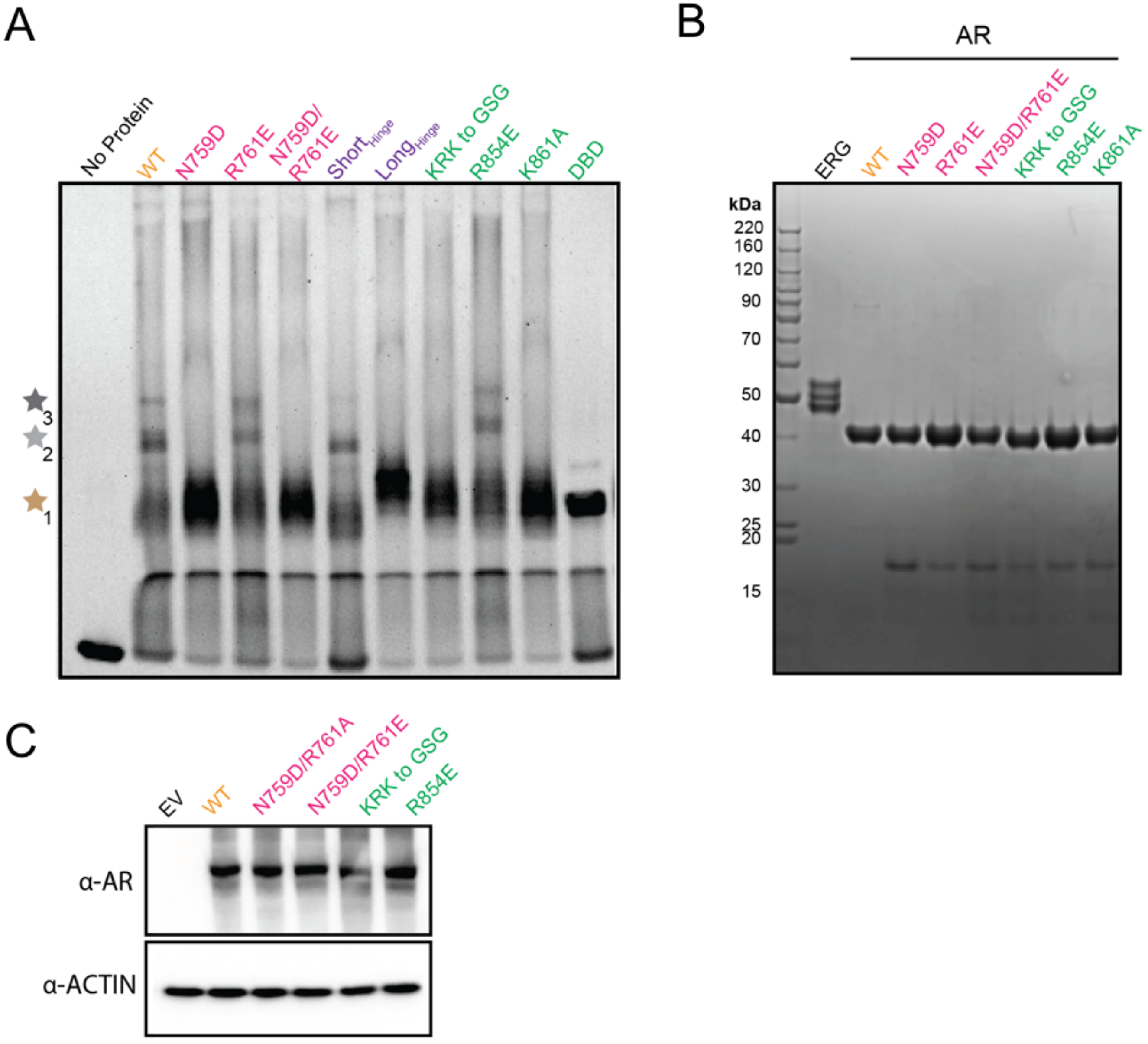

Figure S10. Allosteric surfaces within the AR LBD prime AR activity, Related to Figure 2. (A) DNA gel shift of 50 nM unlabeled ARE duplex DNA using 250 nM of the indicated recombinant protein. 4-20% TBE PAGE stained with Sybr Gold. Stars represent AR-shifted species. (B) Protein gel of final recombinant AR mutant protein preparations used in DNA binding assays. Coomassie-stained 4-12% SDS-PAGE. (C) AR immunoblot of WT and mutants transfected into HEK293 cells for the transactivation assays described in Main Figure 2.

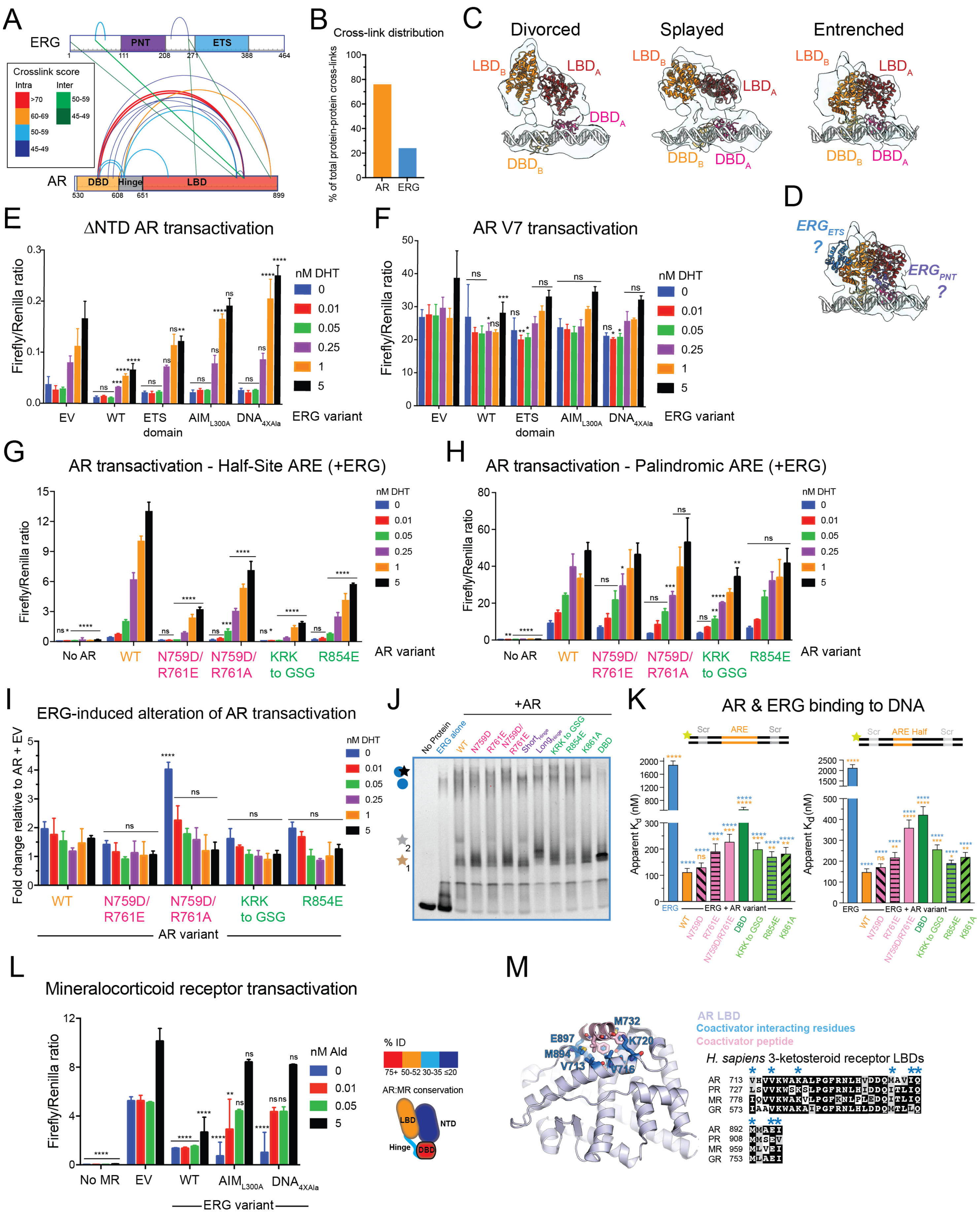
A
ERG
PNT
ETS
Crosslink score
Intra
Inter
>70
60-69
50-59
45-49
50-59
45-49
AR
DBD
Hinge
LBD
B
Cross-link distribution
% of total protein-protein cross-links
AR
ERG
C
Divorced
Splayed
Entrenched
D
E
ΔNTD AR transactivation
Firefly/Renilla ratio
nM DHT
ERG variant
EV
WT
ETS domain
AIM L300A
DNA 4XAla
F
AR V7 transactivation
G
AR transactivation - Half-Site ARE (+ERG)
H
AR transactivation - Palindromic ARE (+ERG)
No AR
N759D/ R761E
N759D/ R761A
KRK to GSG
R854E
AR variant
I
ERG-induced alteration of AR transactivation
Fold change relative to AR + EV
J
+AR
No Protein
ERG alone
K
AR & ERG binding to DNA
Scr
ARE
ARE Half
Apparent Kd (nM)
ERG + AR variant
L
Mineralocorticoid receptor transactivation
nM Ald
No MR
% ID
AR:MR conservation
NTD
M
AR LBD
Coactivator interacting residues
Coactivator peptide
H. sapiens 3-ketosteroid receptor LBDs

Figure S11

Figure S11. ERG interactions mapped to the AR LBD, Related to Figure 3. (A) 2D depiction of DSSO cross-links mapped onto the domain structures of AR and ERG. Cross-links and line thickness are color-coded by likelihood score. (B) Bar graph visualization of the DSSO cross-link distribution in (A). (C) X-ray coordinates of the AR LBD monomer (PDB: 1XOW) (He *et al.*, 2004), the AR DBD dimer (PDB: 1R4I) (Shaffer *et al.*, 2004), and ARE_{35} DNA docked in the cryo-EM density of the Divorced, Splayed, and Entrenched states. Of these three structural models, density that can accommodate the ERG PNT and ETS domains is apparent within the Entrenched model. (D) Hypothetical docking of the PNT (PDB: 1SXE) (Mackereth *et al.*, 2004) and ETS (PDB: 4IRI) (Regan *et al.*, 2013) domains of ERG based on cross-linking in (A,B) and Main Figure 3 and previous *in silico* modeling and mutagenesis assays (Wasmuth *et al*., 2020). (E, F) AR transactivation of ∆NTD AR and splice variant V7 in HEK293 cells on palindromic ARE reporters in the presence of ERG. (G, H) Transactivation of LBD allosteric mutants on the (G) Half site ARE (corresponding to Fig. 3D) and (H) ARE palindromic reporter in the presence of ERG. (I) Data in (H) normalized to AR WT plus empty vector at the specified concentration of DHT. (J) DNA gel shift of 50 nM unlabeled ARE duplex DNA using 250 nM of the indicated recombinant protein in the presence of ERG. 4-20% TBE PAGE stained with Sybr Gold. Stars and circles represent AR- and ERG-shifted species, respectively, with the overlapping symbols indicating a previously characterized supershift unique to AR-ERG complex formation (Wasmuth *et al*., 2020). (K) Fluorescence polarization of AR LBD dimer and DBD interface mutants in the presence of ERG on palindromic (left) and half-site (right) ARE DNA. (L) Top: MR transactivation assay with indicated ERG variants. Bottom: Cartoon depiction comparing sequence conservation between AR and MR. (M) Cofactor interacting residues within the AR LBD are conserved within the 3-ketosteroid NRs. Left: sequence conservation, with cofactor interacting residues indicated with blue asterisks. Right: Cofactor interacting residues within the AR LBD shown as sticks (PBD: 1XOW) (He *et al.*, 2004).

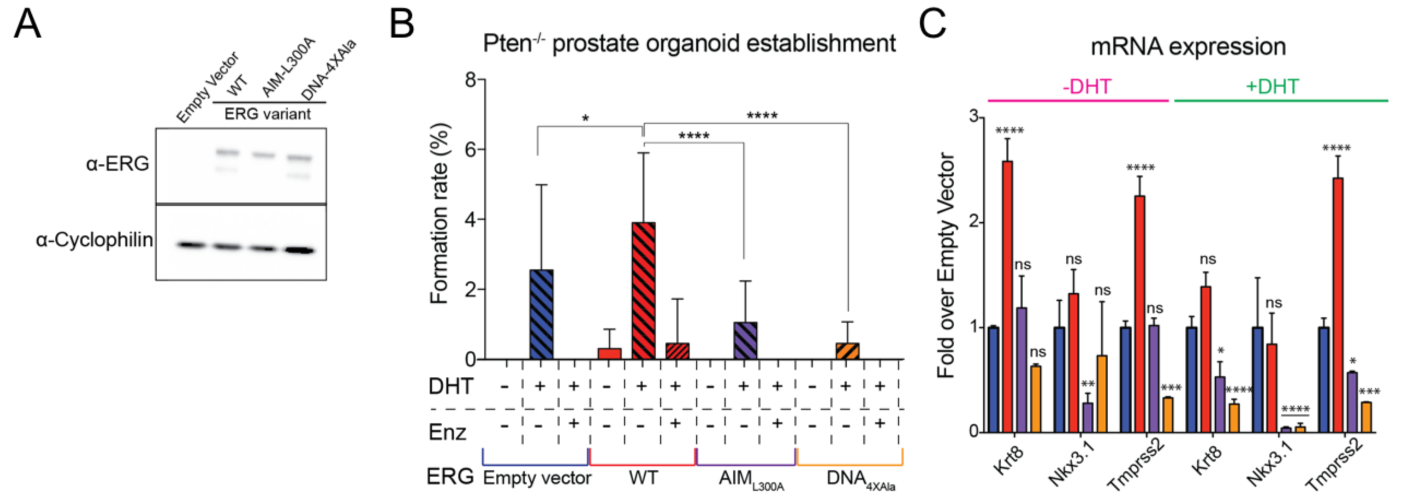

Figure S12. Partly primed AR is more vulnerable to ERG modulation, Related to Figure 3. (A) Immunoblot of ERG protein expression in Pten$^{-/-}$ murine organoids stably integrated with ERG variants. (B) Establishment of Pten$^{-/-}$ organoids with WT and mutant ERG grown in 3D culture in the absence of EGF, with 1 nM DHT or 1 µM Enz added when indicated. (C) Quantitative PCR (qPCR) of organoids from (A, B). mRNA expression of ERG-altered transcripts, including luminal lineage marker Krt8 and AR/ERG co-dependent luminal genes are shown in the absence (left) or presence of DHT (right), conditions that attenuate or promote AR activity, respectively (Karthaus *et al*., 2014; Li *et al*., 2020; Mao *et al*., 2019; Wasmuth *et al*., 2020; Yu *et al*., 2010).

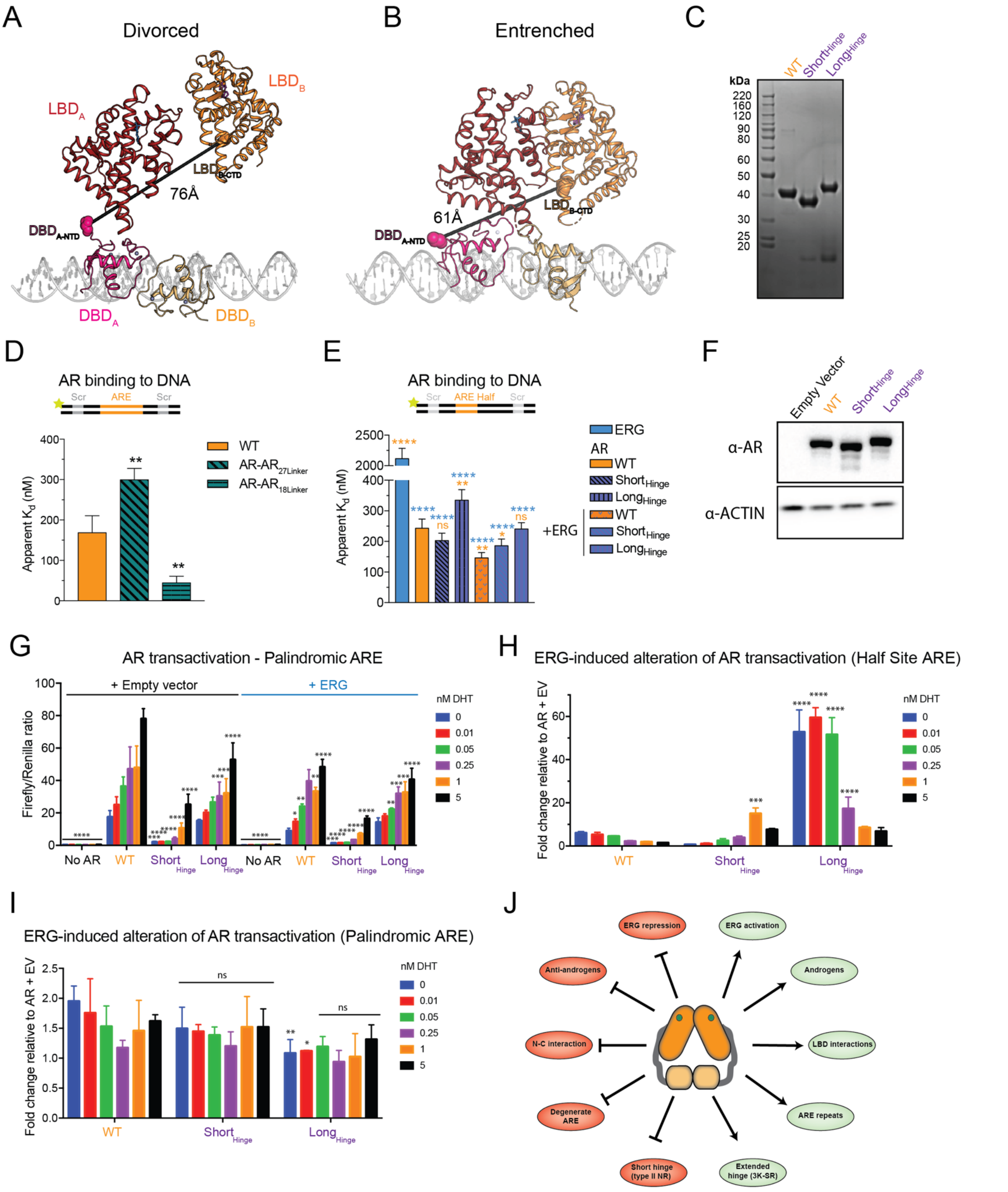
A
Divorced
LBD_A
LBD_B
LBD_B-CTD
76Å
DBD_A-NTD
DBD_A
DBD_B
B
Entrenched
61Å
DBD_A-NTD
LBD_B-CTD
C
WT
Short_Hinge
Long_Hinge
kDa
220
160
120
90
80
60
50
40
30
25
20
D
AR binding to DNA
Scr
ARE
Scr
Apparent K_d (nM)
WT
AR-AR_27Linker
AR-AR_18Linker
E
AR binding to DNA
Scr
ARE Half
Scr
Apparent K_d (nM)
ERG
AR
WT
Short_Hinge
Long_Hinge
+ERG
WT
Short_Hinge
Long_Hinge
F
Empty Vector
WT
Short_Hinge
Long_Hinge
α-AR
α-ACTIN
G
AR transactivation - Palindromic ARE
+ Empty vector
+ ERG
Firefly/Renilla ratio
nM DHT
No AR
WT
Short_Hinge
Long_Hinge
H
ERG-induced alteration of AR transactivation (Half Site ARE)
Fold change relative to AR + EV
nM DHT
WT
Short_Hinge
Long_Hinge
I
ERG-induced alteration of AR transactivation (Palindromic ARE)
Fold change relative to AR + EV
nM DHT
ns
WT
Short_Hinge
Long_Hinge
J
ERG repression
ERG activation
Anti-androgens
Androgens
N-C interaction
LBD interactions
Degenerate ARE
ARE repeats
Short hinge (type II NR)
Extended hinge (3K-SR)

Figure S13

Figure S13. Hinge length impacts DNA binding and AR transcriptional output, Related to Figure 4. (A, B) Coordinate view of the Divorced (A) and Entrenched (B) models and distances in Å between the DBD-C and LBD-N termini of the adjacent protomer. (C) Protein gel of final AR hinge preparations used in DNA binding assays. 4-12% SDS-PAGE stained with Coomassie. (D) Fluorescence polarization of ARE DNA comparing DNA binding of WT AR (ΔNTD) with two protomers of AR fused with a variable linker between the C-terminus of the LBD of protomer A and N-terminus of the DBD of protomer B. The AR-$AR_{18Linker}$ was engineered to mimic a constitutive dimer based on the Entrenched model, in contrast to a variant with an extended linker (AR-$AR_{27Linker}$). (E) Fluorescence polarization of half-site ARE DNA with ERG alone, and AR variants with intact versus shortened and extended hinges in the absence and presence of ERG. (F) Immunoblot of AR protein expression of variants transfected into HEK293 cells for the reporter assays described in Main Figure 4 and this figure. (G) AR transactivation of the ARE consensus reporter in HEK293 cells in the absence or presence of ERG. (H) AR transactivation on the ARE half site reporter in the presence of ERG normalized to WT plus empty vector (Main Figure 4D) at the specified concentration of DHT. (I) AR transactivation on full ARE consensus reporter in the presence of ERG (panel G, this figure) normalized to WT plus empty vector at the specified concentration of DHT. (J) Overview of modes of AR activation and repression highlighted in this study. Activating and repressive signals in green and red, respectively.

Table S1. Cryo-EM statistics, Related to Figure 1.

	Entrenched<br>EMD-25132	Splayed<br>EMD-25133	Divorced<br>EMD-25134
**Data collection & processing**			
Magnification	81,000	81,000	81,000
Voltage (kV)	300	300	300
Electron exposure (e-/Å^2)	61.27	61.27	61.27
Defocus range (μm)	-0.8 - 2.5	-0.8 - 2.5	-0.8 - 2.5
Pixel size (Å)	1.069	1.069	1.069
Symmetry imposed	C1	C1	C1
Initial particle images (no.)	1,683,057	1,683,057	1,683,057
Final particle images (no.)	68,581	53,169	51,454
Map resolution (Å)	11.4	9.1	9.4
FSC threshold	0.143	0.143	0.143
Map sharpening *B* factor (Å^2)	-	-	-
